# The p53 protein plays a central role in the mechanism of action of epigentic drugs that alter the methylation of cytosine residues in DNA

**DOI:** 10.18632/oncotarget.14805

**Published:** 2017-01-24

**Authors:** Arnold J Levine

**Affiliations:** ^1^ Institute for Advanced Study, Princeton, N.J., USA

**Keywords:** epigenetics, P53, Cytosine methylation, AML, Drug resistance

## Abstract

Both normal and cancerous cells, treated with drugs that block cytosine methylation of DNA, are preferentially killed by these drugs when they have p53 mutations and survive if they have a wild type protein. It appears that the wild type p53 protein functions to eliminate cells that undergo large epigenetic alterations and save other cells from death by this drug treatment. This has now been observed in cancerous cells in culture, tumors in animals and tumors in humans. AML cells with p53 mutations in humans treated with decitabine are killed by differentiation or senescense, but then relapse at a high rate becoming drug resistant. The mechanism of resistance to epigenetic drugs in p53 mutant cells, by possibly restoring a wild type p53 gene or restoring a defective p53 pathway, is now an interesting hypothesis to explore.

Eukaryotic DNA is commonly modified by the addition or removal of methylation of cytosine residues at the 5’ position of this base. The presence of the methylation of cytosine can regulate the binding of chromatin modifying enzymes, chromatin structure, alter the transcription of genes associated with this modification, impact the mutation rate of this modified base and regulate DNA repair in these regions of a chromosome. There are three enzymes that catalyze methylation at the 5’ position of cytosine in the dinucleotide CpG (cytosine-phosphate-guanine). DNA-methyltransferase-1 (DNMT1) is the maintenance methytransferase. After DNA replication the template strand may have methylated-CpG while the new strand has the complimentary GpC, and DNA methyltransferase-1 prefers hemi-methylated DNA and adds a methyl-group onto the cytosine residue of the newly replicated strand. DNMT3a and DNMT3b are de novo methytransferases acting in the developing fertilized egg to establish sexually dimorphic genomic imprinting and in stem and progenitor cells to elaborate transcriptional programs leading to the differentiation of cells. These enzymes lay down the epigenetic program that results in the development of organisms and maintains that program in replicating cells of the organism.

Cancerous cells are commonly associated with both genetic alterations and epigenetic alterations, both of which can contribute to the tumorigenic phenotypes. While the genetic mutations are irreversible, the epigenetic modifications of DNA are reversible and a set of drugs have been developed to block the activity of DNMT1 or to function as a substitute for the incorporation of cytosine residues into RNA and DNA (azacytosine and 5’-aza-2’-deoxycytosine) and block the methylation of cytosine residues in DNA. The methylation at certain loci in chromosomes, like the p16 or VHL genes, repress the transcription of these tumor suppressor genes, and it is thought that the drugs that block epigenetic marks in the DNA can now permit the expression of tumor suppressor genes and kill the cancer cells [1]. The first clue that this was too simplistic an observation came from Serrano and his colleagues [2]. Employing 5’aza-2-deoxycytodine to block methylation of cytosine residues in DNA they demonstrated that this drug killed cancer cells in culture that had p53 mutations by triggering apoptosis, but it failed to kill cancer cells or normal cells that had wild type p53 genes and proteins. They showed that this drug entered cells and was incorporated into DNA in both cells with wild type and mutant p53 genes so that the mechanism of cell killing was not due to drug exposure levels. They went on to demonstrate that the wild type p53 gene and protein prevents most of the cells in culture from producing drastically altered epigenetic patterns. The few cells that may have activated a tumor suppressor or triggered p53 mediated apoptosis died. These results suggest that the majority of cells in the drug treated culture were protected from extensive epigenetic changes by the wild type p53 protein no matter whether the cell was a cancerous cell or a normal cell. The wild type p53 protein carried out its proper functions permitting only cells with a normal genome, be it a genetic or epigenetic change, to survive in the culture. This idea that the wild type p53 protein can act as a protector of vitality in drug treated cancer cells as well as a suppressor of cancers arising in the organism is not a new concept [3] and has gained considerable evidence in recent years. We have come to realize that the wild type p53 protein regulates p53 transcriptional programs that repair genetic alterations and prevent epigenetic alterations in cells. The results of Nieto and colleagues [2], were confirmed using cancer cells with p53 mutations, but not wild type p53 proteins, and a large number of different drugs that blocked or altered epigenetic changes in human cancer cells growing as tumors in immune-compromised animals [4]. These observations were reproducible for drugs that inhibited the DNMT1 activity or were faulty incorporators of cytosine residues (5-aza-2-deoxycytosine) into DNA. They were clearly reproduced with several different types of tumors with mutant p53 genes but not with wild type p53 genes. The only difference between the work of Nieto and colleagues [2] and Yi and her colleagues [4] was that in addition to wild type p53 enforcing fidelity by apoptosis, Yi also observed cellular senescence played a role in eliminating some cells in the tumor.

Now these results have been reproduced in humans with two cancers, Acute Myeloid Leukemia (AML) and Myelodisplastic Syndrome (MD) [5]. Employing the drug decitabine or 5’-aza-2-deoxy-cytodine they demonstrated that 21/21 patients with AML and p53 mutations responded to this drug treatment with tumor reductions, while 78 patients with a wild type p53 gene did not respond. Those patients with a p53 mutation commonly had an abnormal karyotype and 20/21 of those patients responded to this drug treatment. Abnormal karyotypes are commonly observed with p53 mutations. The authors speculate that the responses of AML cells with p53 mutations treated with decitabine results from a reduced tumor load due to differentiation of cells under the direction of the mutant p53 protein. Terminal differentiation may be easily confused with or is related to senescence.

Perhaps the most interesting observation made in the Welsh publication [5] is that “Although the presence of Tp53 mutations appears to be associated with a high degree of decitabine sensitivity, relapses in these patients were associated with the outgrowth of a pre-existing subclone in all cases”. It is not clear from the description of the responses to decitabine whether or not each patient achieved a true or complete remission or just a reduction and clearing of leukemic blasts from the marrow. The supplementary materials in the Welsch et al [5] paper demonstrates that only some of the 21 patients that responded to decitibine had p53 variant allele frequencies of greater than 50% consistent with a loss of homozygousity of the wild type allele and only a p53 mutation in the cells. Other AML patients had variant allele frequencies less than 50% suggesting that a wild type p53 allele was retained along with a mutant p53 allele. It would be of some interest to know if these two different classes of AML responded differently to decitabine treatment and if the relapses were different. The fact that there was a response (reducing or clearing leukemic blasts) to decitabine by the 21/21 AML patients with p53 mutations and that response was not durable in every case means that there is a mechanism to overcome the defective p53 pathway in cells that contain mutant p53 proteins. AML cells with wild type p53 proteins do not respond to decitabine. AML cells with a defective p53 pathway lead to epigenetic cell death upon treatment with decitabine. Perhaps the resistance to decitabine treatment observed in the relapses results from the formation of a wild type p53 gene or pathway as is the case observed with some resistance to PARP inhibition in BRCA-1 tumors treated with PARP inhibitors. This novel p53 defective pathway would be expected to select for new clones that resist the ability of the drug to result in extensive epigenetic changes and differentiation, senescence or apoptosis. Resistance to decitabine in cells with p53 mutations has not been reported previously. Examining the mechanism by which this occurs would be an important problem to solve.

A great deal of evidence is already in hand that explores the role of the p53 protein in regulation of epigenetic change. Jackson-Grusby et al [6] employed the floxed loss of the DNMT1 gene from fibroblastic cells in culture, which resulted in the failure to reproduce the methylation pattern in CpG residues of DNA. After two cell divisions, when DNA double strands with no methylation appear, the cells in culture were killed by p53-mediated apoptosis. This required a wild type p53 protein that senses (by an unknown mechanism that likely involves methylation or modifications of the p53 protein) the alterations in the pattern of DNA and likely chromatin methylation resulting in a transcriptional program of cell death. A similar interpretation for the role of the p53 protein in regulating changes in epigenetic marks in a cell is found in the experiments carried out by Yamanaka and his colleagues [7]. The addition and expression of four different transcription factors placed into fibroblasts in cell culture permitted the epigenetic reprograming of a few cells so as to produce induced pluripotent stem cells (iPSC) that were capable of differentiating into many cell types of an embryo. This process was inefficient (about 0.1-1% of the cells) and took a long time to complete (over a period of months). In the absence of p53 however the cells could be reprogramed with only two of the four transcription factors, the efficiency of producing stem cells increased up to 50-80% and the time it took to accomplish this process was reduced to 1-2 weeks [8]. It appears that the efficiency or probability of reprograming epigenetic changes in cells is impacted by the wild type p53 protein. An observation that tempers this hypothesis is the fact that genetic crosses between male and female mice with deleted p53 genes (p53 knock-out mice) give rise to live and normal mice at birth. This requires lots of epigenetic changes in the absence of the p53 protein. However in many different inbred strains of mice about one third of the female mice born are either runted or have an exocephalic (brain outside the cranium) phenotype or developmental abnormalities. Genetic imprinting can often be sexually dimorphic. When the amount of cytosine methylation is examined for the insulin like growth factor gene-2 from the liver of the F-1 offspring of knockout mice (p53−/− mice) the female mice born with genetic abnormalities had a hypermethylation of cytosine residues at the IGF2-H-19 locus. Mice with a wild type p53 gene and protein did not have this hypermethylation phenotype, nor genetic defects [8]. This suggested that p53 knockout female mice demonstrated alterations in epigenetic imprinting in a stochastic or probabilistic fashion.

The observations reviewed here demonstrate a critical role for the wild type p53 pathway in surveillance of the stability of the epigenetic program in a cell. Epigenetic stability in a cell appears to be probabilistic with alterations occurring with age, cell divisions, pathological situations such as cancers, and developmental changes occurring over a lifetime. The epigenetic program can certainly be altered by mutations in one of many genes that modify the DNA, histones and transcription factors regulated by protein or DNA modifications. The stability of the epigenetic program in cells can also be impacted by mutations in the p53 gene. Drugs that alter epigenetic programs and are employed in cancer therapies, function optimally in cells with p53 mutations. Just how the wild type p53 protein blocks these drugs from acting in cells needs to be explored in more detail. It appears that a subset of cells with altered epigenetic programs initiate a wild type p53 transcriptional program that kills those cells. We need to understand the epigenetic changes that trigger p53 activation and a programed cell death. The function of the wild type p53 protein is to insure fidelity by enforcing it with death. It will do this in normal and in cancer cells.
